# Xirp Proteins Mark Injured Skeletal Muscle in Zebrafish

**DOI:** 10.1371/journal.pone.0031041

**Published:** 2012-02-15

**Authors:** Cécile Otten, Peter F. van der Ven, Ilka Lewrenz, Sandeep Paul, Almut Steinhagen, Elisabeth Busch-Nentwich, Jenny Eichhorst, Burkhard Wiesner, Derek Stemple, Uwe Strähle, Dieter O. Fürst, Salim Abdelilah-Seyfried

**Affiliations:** 1 Max Delbrück Center (MDC) for Molecular Medicine, Berlin, Germany; 2 Department of Molecular Cell Biology, Institute of Cell Biology, University of Bonn, Bonn, Germany; 3 Institute for Toxicology and Genetics, Karlsruhe, Germany; 4 University of Southern California Keck School of Medicine, Los Angeles, California, United States of America; 5 Vertebrate Development and Genetics, The Wellcome Trust Sanger Institute, Cambridge, United Kingdom; 6 Leibniz Institute for Molecular Pharmacology, Berlin, Germany; University of Sheffield, United Kingdom

## Abstract

Myocellular regeneration in vertebrates involves the proliferation of activated progenitor or dedifferentiated myogenic cells that have the potential to replenish lost tissue. In comparison little is known about cellular repair mechanisms within myocellular tissue in response to small injuries caused by biomechanical or cellular stress. Using a microarray analysis for genes upregulated upon myocellular injury, we identified zebrafish Xin-actin-binding repeat-containing protein1 (Xirp1) as a marker for wounded skeletal muscle cells. By combining laser-induced micro-injury with proliferation analyses, we found that Xirp1 and Xirp2a localize to nascent myofibrils within wounded skeletal muscle cells and that the repair of injuries does not involve cell proliferation or Pax7^+^ cells. Through the use of Xirp1 and Xirp2a as markers, myocellular injury can now be detected, even though functional studies indicate that these proteins are not essential in this process. Previous work in chicken has implicated Xirps in cardiac looping morphogenesis. However, we found that zebrafish cardiac morphogenesis is normal in the absence of Xirp expression, and animals deficient for cardiac Xirp expression are adult viable. Although the functional involvement of Xirps in developmental and repair processes currently remains enigmatic, our findings demonstrate that skeletal muscle harbours a rapid, cell-proliferation-independent response to injury which has now become accessible to detailed molecular and cellular characterizations.

## Introduction

Among lower vertebrates, many organs have a remarkable regenerative potential. This is particularly evident in the case of the zebrafish heart which can regenerate large injuries by stimulating the proliferation of dedifferentiated cardiomyocytes [1], [2], [3]. Similarly, loss of skeletal muscle can be compensated by the activation and proliferation of muscle stem cells (for a recent review, see [4]). Whereas such regenerative processes require extensive proliferation and occur over days to months, smaller injuries that constantly occur due to excessive exercise and biophysical or cellular stress require more rapid repair mechanisms. A better molecular characterization of such repair processes may provide alternative routes for tissue healing with beneficial implications for humans.

Xin-repeat proteins are striated muscle-specific actin-binding multi-adaptor proteins that interact with sarcomeric proteins or F-actin associated proteins and localize to the intercalated discs (ICD) of cardiomyocytes or to the myotendinous junction (MTJ) of skeletal muscle cells [5], [6], [7], [8], [9], [10], [11]. Xirp1 was found to be upregulated in mouse models of hypertension [12], [13], in other mouse models based on eccentric exercise [14], [15], in a spontaneous mouse mutant with regenerating muscle tissue [15], [16], and in the dystrophic zebrafish mutant *runzel* [17]. Currently, a coherent functional understanding of Xirp family proteins is lacking. Whereas cXin, the only Xirp family member present in chicken, has been proposed to regulate cardiac morphogenesis [11], both mammalian Xirps, Xirp1 and Xirp2, have been implicated in hypertrophic cardiomyopathy in humans and mice [18], [19], [20], [21]. Here, we show that, although not being functionally essential during development, Xirp1 is strongly induced within injured skeletal muscle in the zebrafish embryo. Because Xirp1^+^ injured muscle can recover in the course of hours and does not involve cell proliferation, our study may have potential implications for understanding the molecular and cellular biology of myocellular repair in humans.

## Results

### *xirp1* is upregulated upon myocellular injury

**Figure 1:**
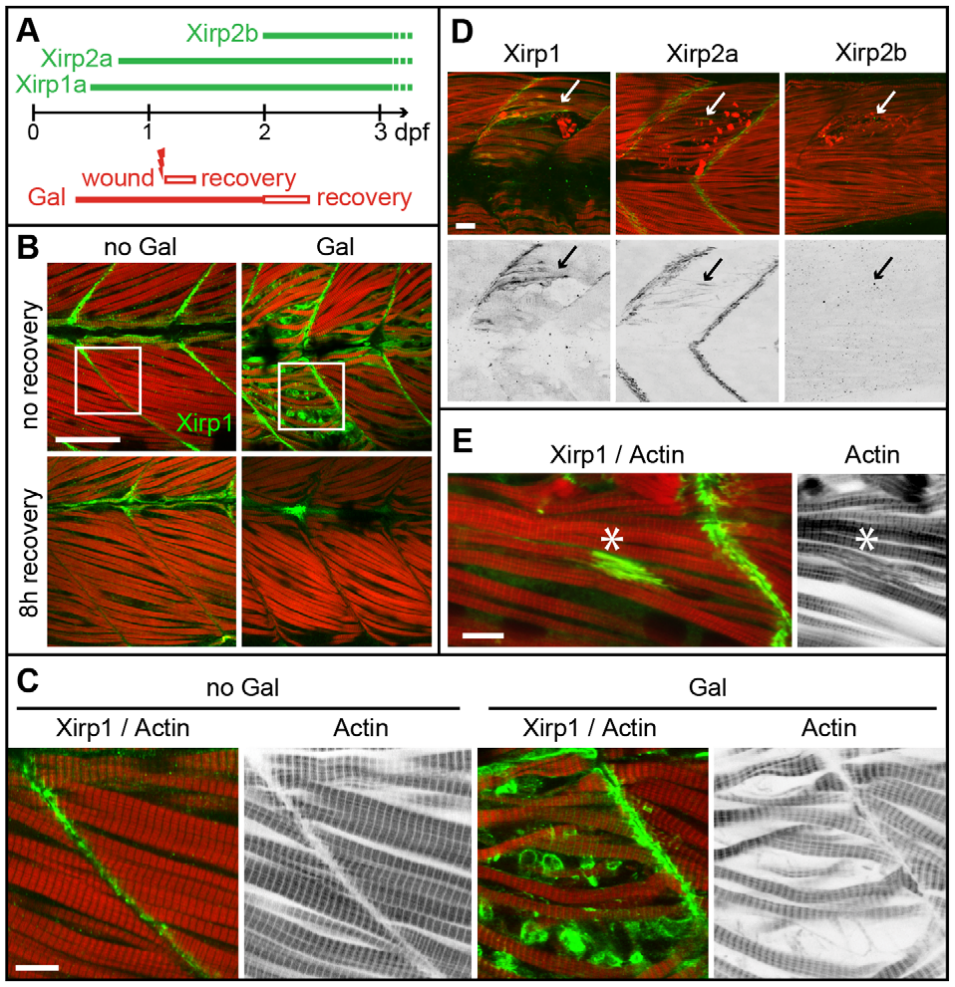
Expression and localization of Xirps within Galanthamine- and laser-induced myocellular wounds. (A) Schematic diagram summarizing the temporal order of Xirp expression within somitic muscle and in myocellular wounding assays. (B) Galanthamine (GAL) treatment between 80% epiboly and 2 dpf causes severe disruptions of somitic muscle organization and myofibrillar disarray (red: Actin) in 2 dpf zebrafish embryos. Notably, Xirp1 (green) is strongly expressed and localizes within cells most strongly disrupted by the treatment. These effects are completely reversible within several hours of recovery. Scale bar: 50 µm. (C) Details from inserts indicated in B (green: Xirp1; red: Actin). Scale bar: 10 µm. (D, E) Similarly, laser-induced muscle injury induces ectopic Xirp1 and Xirp2a localization to damaged myofibrils. In comparison, Xirp2b is not yet expressed at 33 hpf. Green: Xirp1, Xirp2a or Xirp2b; red: Actin. Arrows indicate the position of laser-induced injury within somitic tissue. Scale bars: 10 µm. Embryos were injured at 27 hpf (D) or 29 hpf (E) and fixed at 33 hpf (D) or 31.5 hpf (E), respectively.

In a search for proteins involved in zebrafish embryonic muscle repair, we performed a transcriptome analysis in a pharmacologically induced model of muscle injury. Zebrafish embryos treated with the acetylcholinesterase (AChE) inhibitor Galanthamine (Gal) develop a severe disarray of somitic muscle organization after 48 hours post fertilization (hpf) due to an over-activation of muscle cells by the accumulation of the neurotransmitter acetylcholine [22]. We assessed the efficacy of the treatment based on the impaired motility of embryos which strongly correlated with a disarray of myofibrils within skeletal muscle cells (Fig. 1A,B,C). The effects of Gal-treatment were reversible within several hours of recovery although the cellular and molecular mechanisms involved in this repair process are not known (Fig. 1B). One of the most strongly affected genes upon Gal-treatment was the three-fold upregulated *xirp1* gene. In comparison, no other muscle-specific gene was significantly upregulated except Desmin (Table 1). This finding was verified by immunohistochemistry using a zebrafish-specific anti-Xirp1 antibody in Gal-treated embryos. Immediately after Gal-treatment, skeletal muscle cells displayed disordered arrays of myofibrils that contained high levels of Xirp1 protein (Fig. 1B,C). Within 8 hours of recovery, Gal-induced lesions had recovered and ectopic Xirp1 was not further detectable (Fig. 1B).

**Table 1:** Transcriptome analysis for genes regulated by Galanthamine treatment in zebrafish.

Compugen Serial Number	FC at 56 hpf	FC at 72 hpf	Gene name
CGENZEB_456010175_0	6,89	8,57	SOCS-3a
CGENZEB_456005792_0	4,83	2,00	zgc:92851
**CGENZEB_456004831_0**	**3,32**	**3,22**	**cardiomyopathy associated 1 (xirp1)**
CGENZEB_456004003_0	2,53	3,94	SOCS-3b
CGENZEB_456009506_0	2,37	8,46	fibronectin 1b
CGENZEB_456005408_0	2,19	1,71	zgc:92069
CGENZEB_456003428_0	2,14	2,43	activating transcription factor 3
CGENZEB_456009990_0	2,06	3,67	complement component 6
CGENZEB_456006757_0	2,05	1,88	Q6PBK2_BRARE (GenBank: AAH59678.1)
CGENZEB_456002498_0	2,00	2,19	coagulation factor XIII, A1 polypeptide
CGENZEB_456003229_0	1,62	2,07	phosphoenolpyruvate carboxykinase 1
CGENZEB_456000995_0	1,61	3,38	desmin
CGENZEB_456001821_0	1,44	2,23	uncoupling protein 4

Summary table listing those genes that are most strongly upregulated in the acetylcholinesterase inhibitor Galanthamine-induced model of muscle injury. For treatment, zebrafish embryos were continuously incubated with Galanthamine between the end of gastrulation and several days of development. Comparisons of fold changes (FC) at 56 hpf and 72 hpf are shown for those genes showing the strongest expression changes. *xirp1* is more than three-fold upregulated at both time-points under conditions of myocellular injury.

### Zebrafish Xirp proteins have overlapping but distinct expression patterns

**Figure 2:**
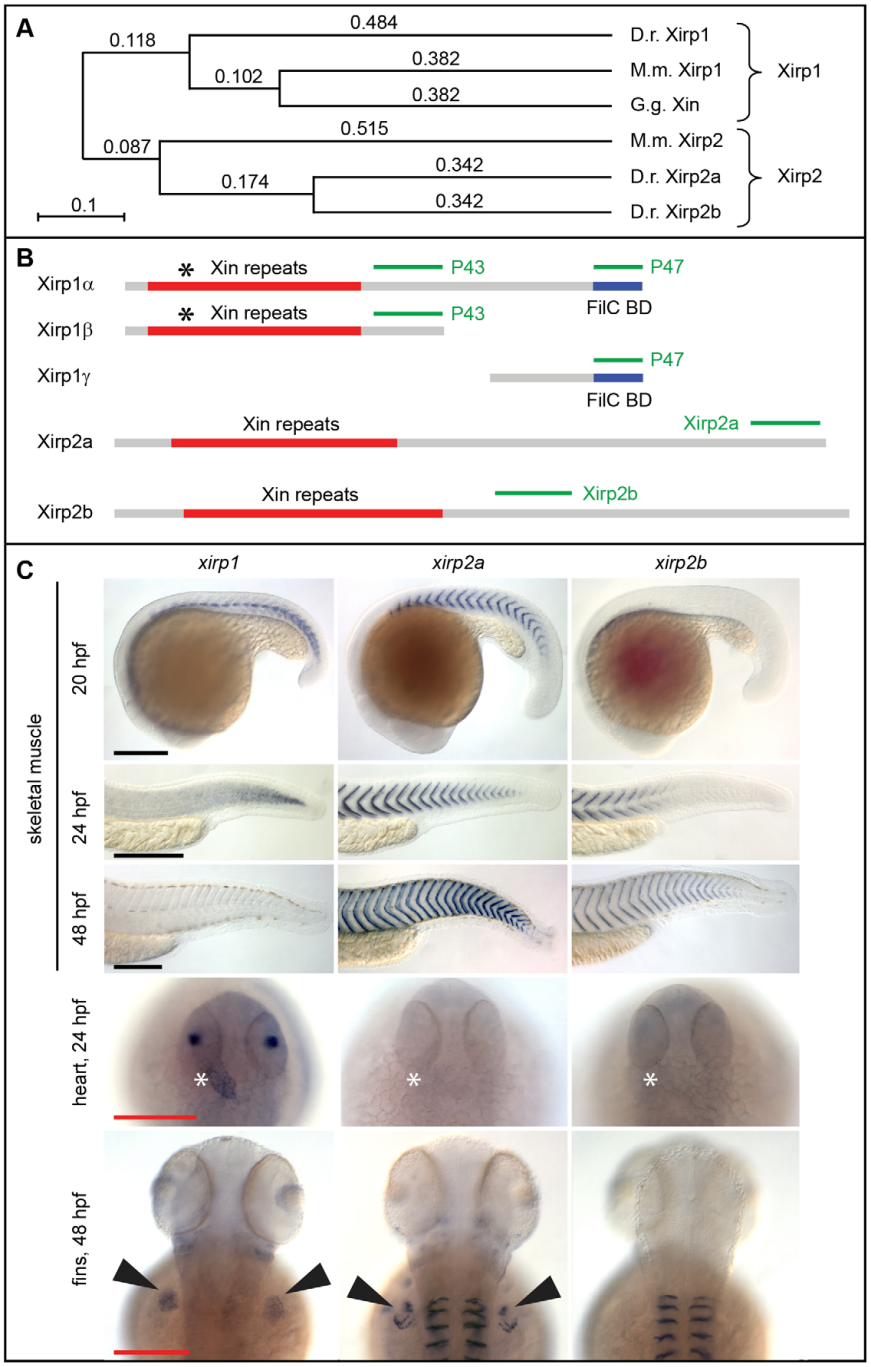
Structure of the zebrafish Xirp family and gene expression patterns. (A) Phylogenetic analysis of Xirp family members. The zebrafish Xirp family comprises three members which are orthologous to their two mammalian counterparts. Phylogenetical distances calculated according to the ClustalW slow/accurate method are indicated. *Danio rerio (D.r.); Gallus gallus (G.g.); Mus musculus (M.m.).* (B) As for mammalian Xirp1, intraexonic splicing of zebrafish *xirp1* results in three alternative isoforms: *xirp1α* (6894 bp), *xirp1β* (4452 bp), *xirp1γ* (2006 bp). Xirp1β is identical to the N-terminus of Xirp1α and harbours Xin-repeats; Xirp1γ is identical to the C-terminus of Xirp1α and harbours a Filamin C binding domain (FilC BD). Antibodies specific against isoforms Xirp1α/β (P43) and Xirp1α/γ (P47) were generated. Black asterisks indicate the position of protein truncation in the *xirp1^sa0046^* mutant. Concerning Xirp2a and Xirp2b, differential splicing leads to various isoforms classified as long (containing Xin-repeats) or short (lacking a large exon encoding Xin-repeats and resulting in the encoding of a LIM domain). Here, we represented schematically the long isoforms and their respective antibody. (C) Summary panel of *xirp* gene family expression with whole-mount *in situ* hybridization probes designed to exclusively cover the long isoforms of these genes. *xirp1* has the earliest and widest expression of all three *xirp* genes. After initial expression within adaxial cells during somitogenesis, *xirp1* expression decreases within somitic tissue after 24 hpf. *xirp1* is the only *xirp* family member harboring Xin-repeats that is expressed within the heart during cardiogenesis. In comparison, *xirp2a* and *xirp2b* mRNAs are strongly expressed within somitic muscle after 24 hpf where they localize to the somite boundaries. Both *xirp1* and *xirp2a* are expressed within other types of skeletal muscle including those of the fin buds, whereas *xirp2b* expression remains restricted to somitic muscle. Asterisks indicate the heart; arrowheads point at fin buds. Black scale bars: 250 µm, red scale bars: 200 µm.

The zebrafish *xirp* gene family comprises three members which are orthologous to their two mammalian counterparts *Xirp1* and *Xirp2* (Fig. 2A) [23]. As in mammals, zebrafish *xirp1* is encoded by a large exon and can yield three isoforms as a result of intraexonic splicing (Fig. 2B). The gene structure of both *xirp2a* and *xirp2b* consists of several short exons (*xirp2a*: exon 1–8; *xirp2b*: exon 1–7) preceding a major large exon encoding the Xin-repeats (*xirp2a*: exon 9; *xirp2b*: exon 8), and followed by two short exons (*xirp2a*: exon 10–11; *xirp2b*: exon 9–10). Evolutionarily conserved splicing mechanisms result in large isoforms with Xin-repeats and short isoforms lacking the Xin-repeats, but encoding a LIM domain created by splicing together the short exons. Our sequence analyses have revealed the expression of both large and short isoforms. However, the short isoforms lack the characteristic Xin-repeats.

**Figure 3:**
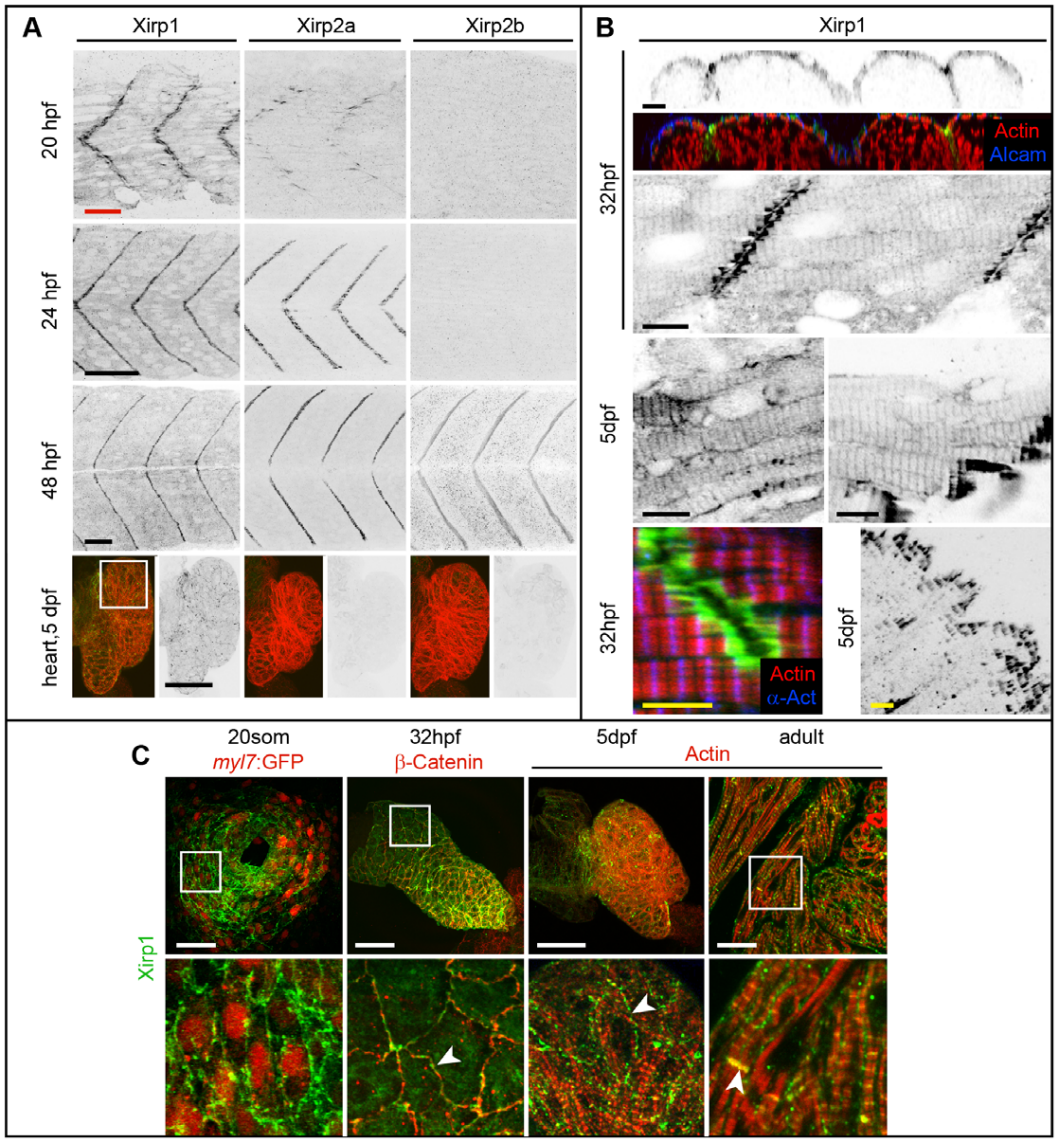
Expression and localization of Xirp proteins during skeletal and cardiac muscle development in WT. (A) Xirp expression patterns overlap within skeletal muscle between 20–48 hpf. In comparison, only Xirp1 is expressed within the cardiac tissue at 5 dpf. Green: Xirp1, Xirp2a or Xirp2b; red: Actin. Red scale bar: 25 µm, black scale bars: 50 µm. (B) Top panel shows cross section planes of confocal z-scan projections through somitic tissue. Note that Xirp1 (green) is most strongly expressed within the outer layer of slow muscle cells, as marked by Alcam (blue) expression (red: Actin). Whereas most of Xirp1 localizes in a staircase-like pattern to the MTJ, some protein is also detectable in a repetitive pattern at the z-discs of myofibrils and at the lateral plasma membrane, depending on the tissue. Different skeletal muscle types are shown at 5 dpf, whereas somitic muscle is shown at 32 hpf. Green: Xirp1; red: Actin; blue:α- Actinin. Black scale bars: 10 µm, yellow scale bars: 5 µm. (C) Expression and localization of Xirp1 during cardiogenesis and in adult cardiac tissue. Bottom row represents higher magnification images (from inserts, if there is a white box on the top picture) with details on Xirp1 subcellular localization at the ICD of cardiomyocytes (arrowheads). Green: Xirp1; red: *myl7*:GFP, β-Catenin or Actin. Scale bars: 50 µm.

We performed whole-mount *in situ* hybridizations and raised antibodies against Xirp1, Xirp2a and Xirp2b to determine their expression and subcellular localization patterns. The expression of *xirp1*, *xirp2a* and *xirp2b* starts in a precise temporal order and mirrors the progression of skeletal muscle cell differentiation (Fig. 2C; Fig. 3A). *xirp1* has the earliest and broadest expression and is present in all striated muscles (Fig. 2C). On the protein level, Xirp1 localizes to the MTJ and the Z-discs and is continuously expressed in all striated muscle from earliest developmental stages until adulthood (Fig. 3). Importantly, Xirp1 is the only zebrafish Xirp protein containing Xin-repeats that is expressed in the heart (Fig. 2C; Fig. 3A,C). In comparison, *xirp2a* and *xirp2b* are expressed later, their expression is restricted to skeletal muscle or somitic muscle, respectively, and their mRNAs are localized to the MTJ (Fig. 2C). Xirp2a and Xirp2b proteins localize exclusively to the MTJ (Fig. 3A).

### Xirp1 and Xirp2a localize along injured myofibrils

In contrast to *xirp1*, neither *xirp2a* nor *xirp2b* were represented in our initial transcriptome analysis of myocellular injury. To characterize the mode of myocellular repair in more detail, as well as to assess expression levels and localization of all three Xirps within myocellular lesions, we used a micropoint laser to induce micro-injuries with great temporal and spatial precision (Fig. 1A,D,E; movie S1). We first assayed by immunohistochemistry the expression of Xirps in response to laser-induced muscle injury. Indeed, the localization of Xirp1 and Xirp2a was altered in response to myocellular injury at 32 hpf when Xirp2b was not yet expressed (Fig. 1A,D). Most strikingly, Xirp1 and Xirp2a were enriched along non-striated portions of injured myofibrils (Fig. 1D,E). After laser-induced micro-injury, there was a complete recovery from tissue damage within 2–8 hours. This observation was indicative of myocellular repair that is considerably more rapid than expected for regenerative processes that involve cell proliferation.

### Xirp1 marks wounded skeletal muscle cells prior to *de novo* cell proliferation

**Figure 4:**
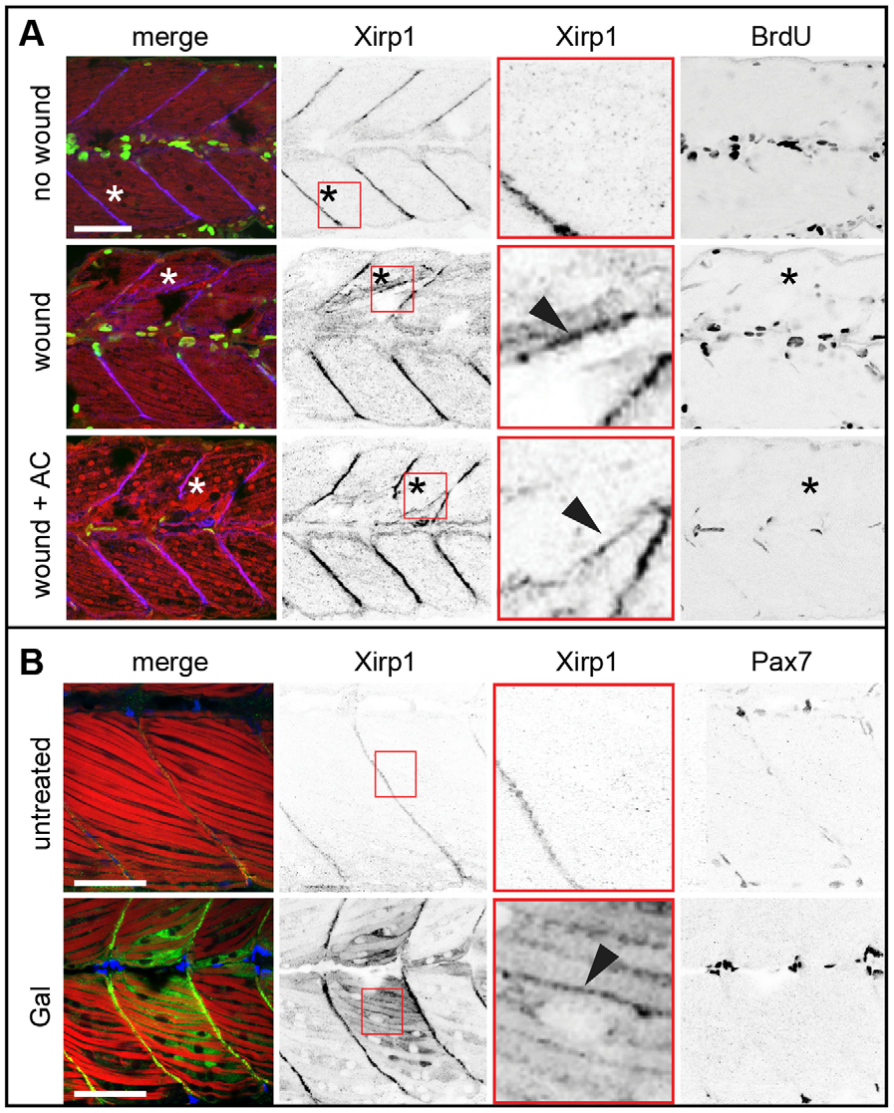
Xirp1 marks wounded skeletal muscle cells prior to *de novo* cell proliferation. (A) Laser-induced myocellular injuries in 33 hpf old zebrafish embryos after BrdU pulse labeling (between 24–33 hpf). Embryos were wounded at 31 hpf and left to recover for 2 hours. Xirp1^+^ tissue is devoid of BrdU^+^ proliferative cells. Treatment of laser-induced myocellular injuries with the proliferation inhibitor Aphidicolin (together with BrdU between 24–33 hpf) does not affect expression and localization of Xirp1 within damaged tissue. The efficacy of the Aphidicolin treatment is evident from strongly reduced BrdU labelling. Asterisks indicate the position of laser-induced injury within damaged tissue. Red inserts show Xirp1 localization within damaged tissue. Green: BrDU; red: Actin; blue: Xirp1. (B) Consistent with the lack of proliferating cells within Xirp1^+^ damaged tissue, the distribution of Pax7^+^ external cells is not changed compared to control conditions. Arrowheads mark ectopic Xirp1. Red inserts show Xirp1 localization within damaged tissue. Green: Xirp1; red: Actin; blue: Pax7. All scale bars: 50 µm.

Next, we aimed at analyzing whether upregulation of Xirp1 within damaged tissue correlated with contributions of proliferating myogenic progenitor cells. To this end, embryos were laser-injured and subsequently analyzed either with 5-bromodeoxyuridine (BrdU)-labelling to detect proliferating cells or with an antibody against Pax7 which detects external cells, a type of muscle stem cells found in zebrafish embryos [24], [25]. As expected for such a rapid repair mechanism, within 2 hours of recovery, laser-injured regions contained Xirp1^+^ cells all of which were BrdU^−^ (Fig. 4A). Thus, repair of the injured tissue occurred in the absence of cells that had recently undergone DNA synthesis. In comparison, laser injuries induced some proliferation within damaged superficial cell layers (data not shown).

To further substantiate this finding, we next used Aphidicolin to inhibit cell proliferation [26]. Continuous Aphidicolin-treatment during recovery after laser-induced micro-injury neither prevented localization of Xirp1 to injured myofibrils nor tissue repair (Fig. 4A). To assess the efficiency of wound healing, we performed histochemistry using rhodamine phalloidin to visualize disrupted myofibrils and found that less than half of the wounds were still detectable 6 hours after injury, as in wild-type (WT) [number of open, visible wounds/number of injuries: WT, (n = 8/32; 25%); Aphidicolin-treated WT, (n = 17/40; 42.5%); no significant difference according to t-test: p-value 0.2149]. Moreover, Xirp1^+^ cells did not overlap with Pax7^+^ cells upon Gal treatment (Fig. 4B). After wound healing, Xirp1 was not further detectable with ectopic localization. Together, these findings suggested that Xirp1 marks injured skeletal muscle which can recover in the absence of significant cell proliferation.

**Figure 5:**
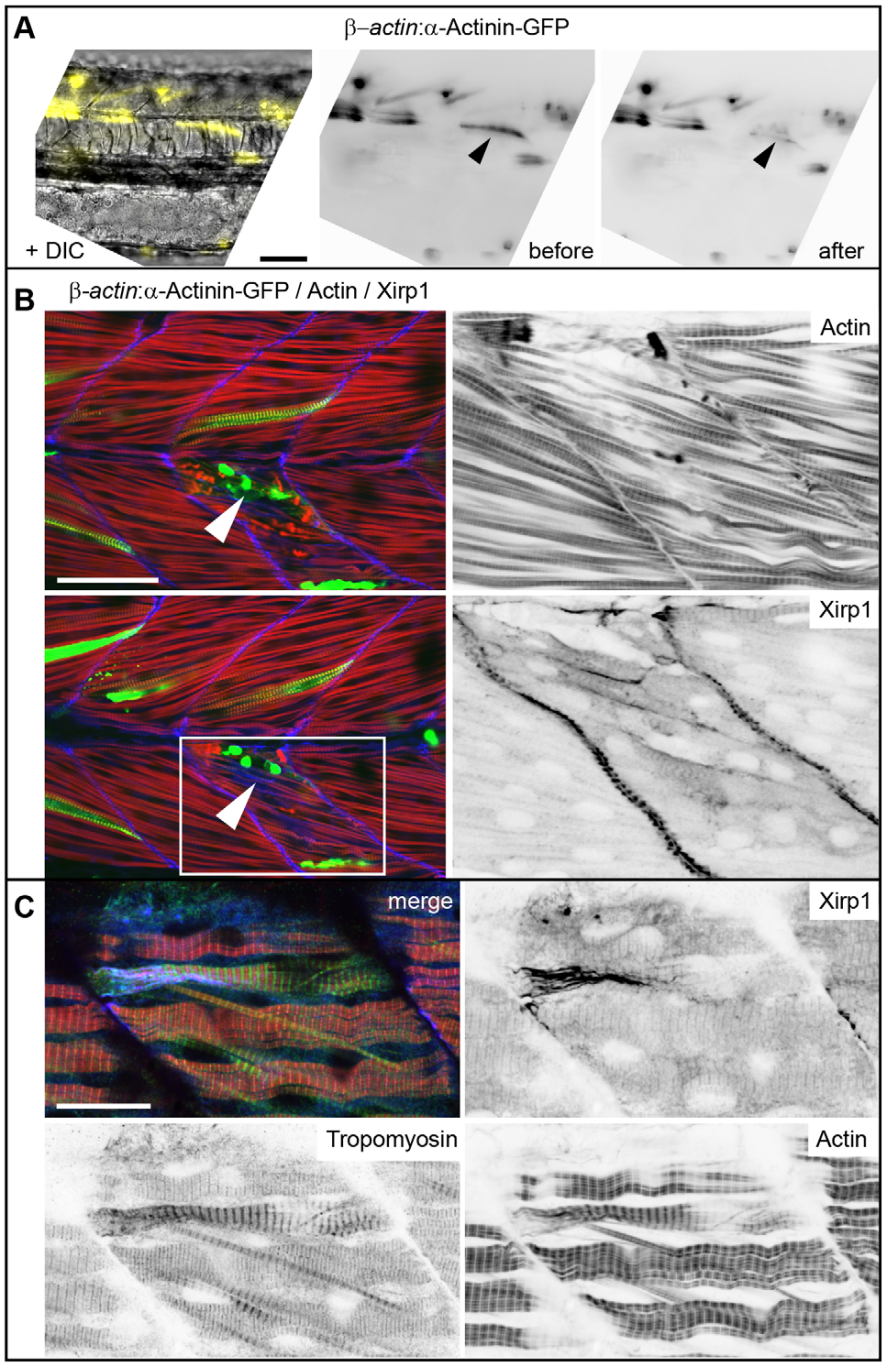
Clonal analysis of laser-induced myocellular injuries. (A–B) Laser-injury and recovery within the same embryo. (A) Left: DIC image superimposed with image of individual muscle cells marked by Tg[*βactin:α-actinin-gfp*] expression (false colored in yellow). Right: laser-injury was performed by targeting one of the α-Actinin-GFP positive muscle cells. Immediately upon laser-injury, α-Actinin-GFP expression within the damaged cell is diminished (arrowheads). Scale bar: 50 µm. (B) Confocal images of two z-scan planes of an immunohistochemical staining show strong Xirp1 expression within 2.5 hours after laser injury in muscle cells directly adjacent to the targeted cell that was most severely affected by the laser-injury (arrowheads). Pictures on the right are details from the insert (white box on the bottom left picture). Green: *βactin*:α-Actinin-GFP; red: Actin; blue: Xirp1. Scale bar: 50 µm. (C) Confocal z-scan projection of an immunohistochemical staining 5 hours after laser-induced injury reveals that strong Xirp1 expression correlates with a pattern of Tropomyosin distribution that is different from unaffected regions of the somite. Green: Tropomyosin; red: Actin; blue: Xirp1. Scale bar: 20 µm.

Another possibility for skeletal muscle recovery could be that cellular rearrangements of cells neighboring localized lesions may be involved. To investigate this possibility, we genetically labeled single muscle cells with a Tg[*βactin:α-actinin-GFP*] expression construct and performed laser-assisted micro-injuries only on those cells expressing the fusion protein (Fig. 5). This analysis revealed that Xirp1 was expressed within those cells that had directly been targeted by the laser and within their direct neighbors (Fig. 5A,B). Immunohistochemical analysis of injury sites using an antibody against Tropomyosin revealed that strong Xirp1 expression correlates with a striation pattern of Tropomoysin that is different from unaffected regions of the somite and which indicates that myofibrillar remodeling is occurring within injured cells (Fig. 5C) [26]. Since Xirp1 was detected on non-striated portions of myofibrils, correlated with a unique pattern of Tropomyosin, and was expressed directly within injured cells or within cells neighboring the injury site, Xirp1 appears to be part of a myocellular injury response that involves myofibrillar remodelling.

### Myocellular repair occurs in the absence of Xirp1 and Xirp2a

**Figure 6:**
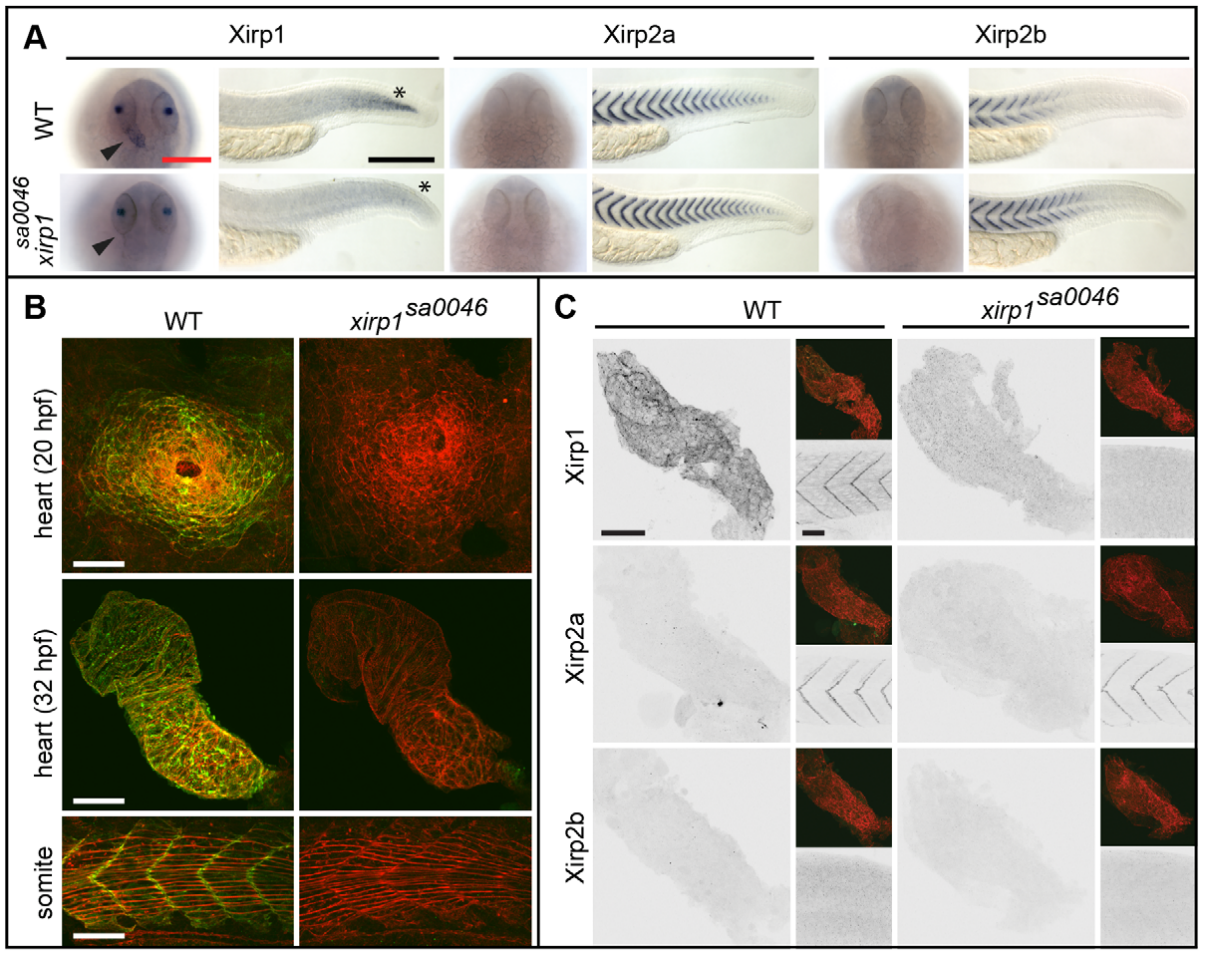
Normal cardiogenesis and skeletal muscle development in the *xirp1^sa0046^* mutant. (A) Whole-mount *in situ* hybridization of *xirp1*, *xirp2a* and *xirp2b* on 24 hpf WT and *xirp1^sa0046^* mutants reveal a loss of *xirp1* expression in heart and muscle of *xirp1^sa0046^* mutants. In contrast, neither *xirp2a* nor *xirp2b* expression are affected by lack of *xirp1*. In particular, neither *xirp2a* nor *xirp2b* are upregulated in the hearts of *xirp1^sa0046^* mutants. Arrowheads indicate the position of the heart tube. Asterisks indicate the tip of the tail. Red scale bar: 200 µm, black scale bar: 250 µm. (B) Lack of *xirp1* mRNA expression corresponds with the complete absence of Xirp1 (green) within the 20 hpf heart cone, 32 hpf heart tube, and 21 hpf skeletal muscle. The Actin counterstaining (red) demonstrates that cardiogenesis and skeletal muscle organization at these stages is comparable between WT and *xirp1^sa0046^* mutants. Scale bars: 50 µm. (C) Immunohistochemical stainings for Xirp1, Xirp2a and Xirp2b on 24 hpf WT and *xirp1^sa0046^* mutant hearts show complete absence of Xirp2a and Xirp2b. Top inserts depict overlay images of Actin (red) and Xirp1, Xirp2a or Xirp2b (green). Within the WT somitic muscle, both Xirp1 and Xirp2a are expressed whereas Xirp1 is absent in *xirp1^sa0046^* mutant somitic tissue (bottom inserts). Scale bars: 50 µm.

**Figure 7:**
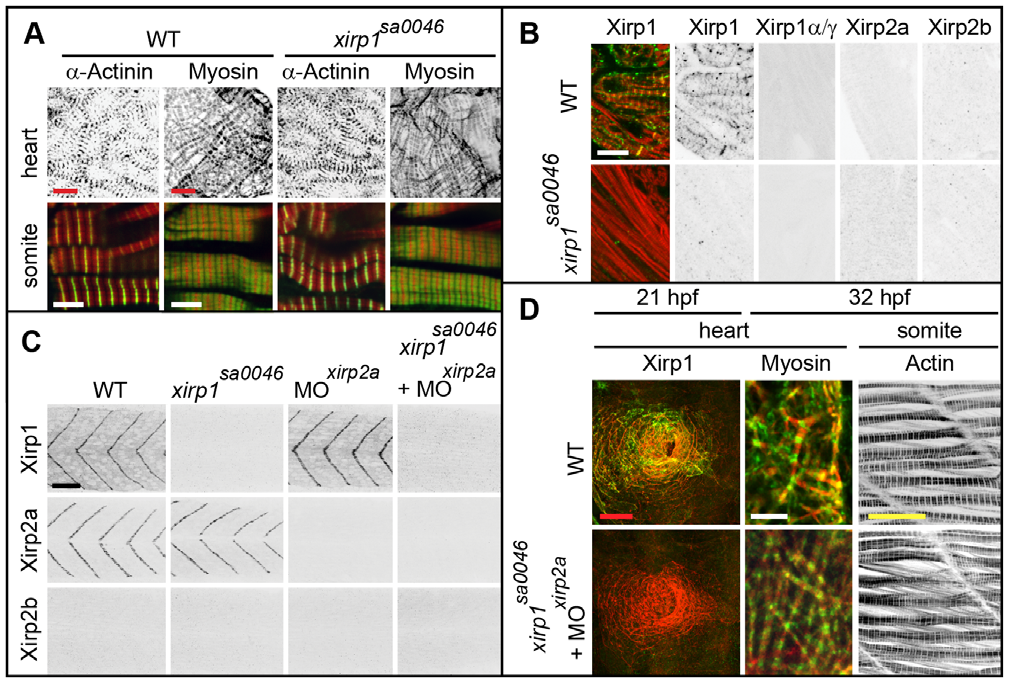
Normal cardiogenesis and myofibrillogenesis in the absence of all Xirps. (A) Neither cardiac nor skeletal muscle sarcomeric organization of myofibrils is affected in 48 hpf *xirp1^sa0046^* mutants. Green: α-Actinin or Myosin; red: Actin. Red scale bars: 10 µm, white scale bars: 5 µm. (B) Sectioned adult cardiac tissue reveals that neither Xirp2a nor Xirp2b are expressed to compensate for the loss of Xirp1. Also, the *xirp1^sa0046^* mutant lacks all three Xirp1 isoforms. Therefore, Xirps are not required for development or maintenance of cardiac tissue. Green: Xirp1; red: Actin. Scale bar: 10 µm. (C) Complete absence of all Xirps within skeletal muscle prior to 24 hpf in *xirp1^sa0046^*/*xirp2a*MO mutant/morphants. Localization of Xirp1 is not affected in *xirp2a* morphants and, conversely, Xirp2a localization is normal in *xirp1^sa0046^* mutants. Scale bar: 50 µm. (D) Complete loss of Xirps in *xirp1^sa0046^*/*xirp2a*MO mutant/morphants does not impair early cardiogenesis (at 21 hpf) or myofibrillogenesis (at 32 hpf). Green: Xirp1 or Myosin; red: Actin. Red scale bar: 50 µm, white scale bar: 5 µm, yellow scale bar: 20 µm.

**Figure 8:**
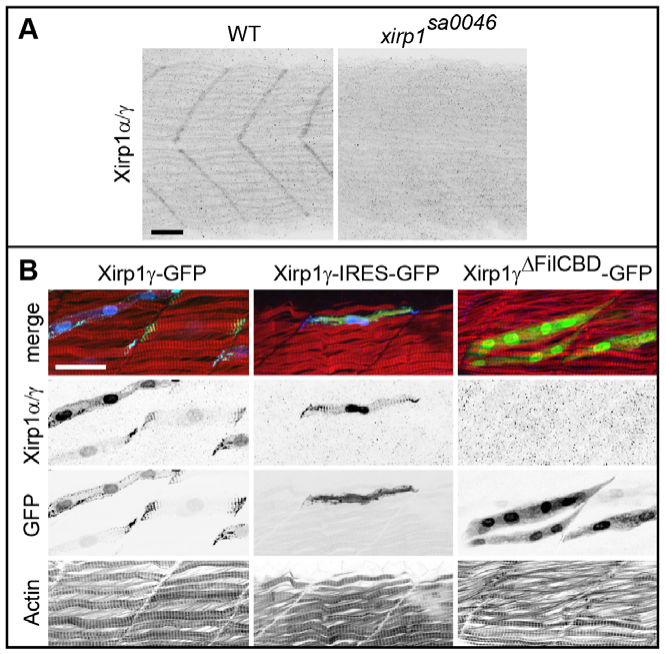
Characterization of the P47 antibody. (A) Myotendinous junction labeling by the P47 antibody which recognizes Xirp1α/γ is absent in *xirp1^sa0046^* mutant embryos at 24 hpf. This staining was performed upon mild fixation. (B) Clonally expressed Xirp1γ is sensitively detected upon standard fixation conditions by the P47 antibody within somitic muscle tissue. Truncated Xirp1γ^ΔFilCBD^-GFP which lacks the Filamin C binding domain including the P47 epitope is not detected by the antibody. Under standard fixation conditions, Xirp1α/γ is not detected at the myotendinous junctions. Green: GFP or Xirp1γ-GFP fusion protein; red: Actin; blue: Xirp1α/γ. All scale bars: 25 µm.

**Figure 9:**
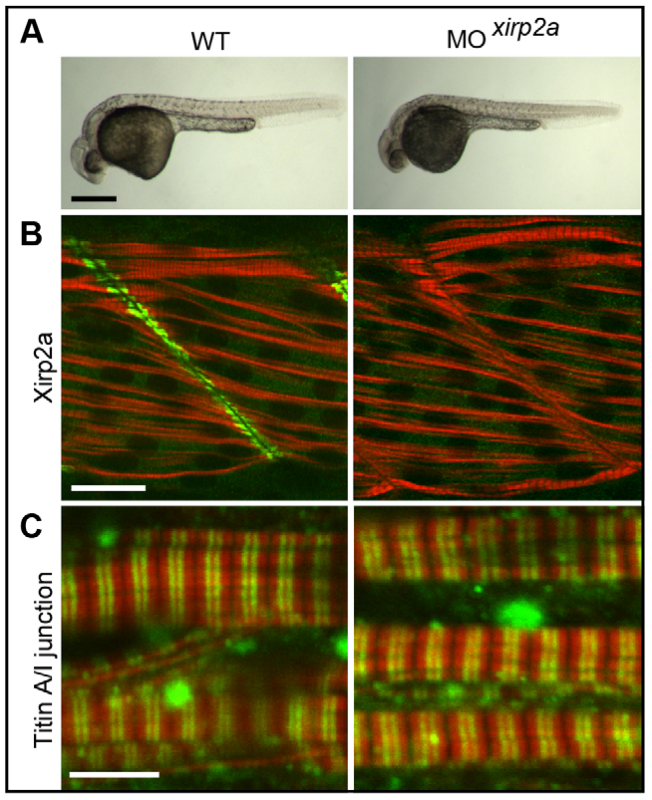
Efficient Morpholino antisense oligonucleotide-mediated knock-down of *xirp2a* does not affect myofibrillogenesis. (A) Phenotypically, *xirp2a* morphants are indistinguishable from WT embryos at 24 hpf. Scale bar: 250 µm. (B) Efficient gene knock-down of *xirp2a* is assessed by immunohistochemistry at 24 hpf. Green: Xirp2a; red: Actin. Scale bar: 20 µm. (C) Complete loss of Xirp2a does not affect myofibrillogenesis and correct sarcomeric organization of skeletal muscle as determined by immunohistochemistry using an antibody against the A/I junction epitope of Titin and rhodamine phalloidin to label sarcomeric Actin at 24 hpf. Green: Titin; red: Actin. Scale bar: 5 µm.

To elucidate the functional role of Xirp1 in myocellular repair, we obtained the *xirp1^sa0046^* mutant allele which causes a premature stop codon replacing Lys_271_. Expression of *xirp1* mRNA was not detectable in cardiac or somitic tissue of *xirp1^sa0046^* mutants, suggesting that the mutation is a null allele causing a complete loss of Xirp1 protein (Fig. 6A). Consistent with the absence of mRNA expression, immunohistochemical analyses with isoform-specific antibodies confirmed the complete lack of all three expected Xirp1α/β/γ protein isoforms in cardiac and skeletal muscles (Fig. 6B,C; Fig. 7B,C; Fig. 8). Unexpectedly, *xirp1^sa0046^* mutants did not exhibit any obvious developmental defects and were adult viable and fertile (see below). Similarly, MO-mediated knock-down of *xirp2a* [27] in WT as well as in *xirp1^sa0046^* mutant embryos did not visibly affect embryonic development (see below; Fig. 7C,D; Fig. 9).

Next we analyzed recovery of skeletal muscle upon laser-injury in *xirp1^sa0046^*/*xirp2a*MO mutant/morphant embryos. Myocellular injuries efficiently recovered within 6 hours with normal temporal dynamics after laser-injury [number of wounds recovered/number of wounds: WT (n = 11/20; 55%); *xirp2a* morphant (n = 12/20; 60%); *xirp1^sa0046^* mutant (n = 14/20; 70%); *xirp1^sa0046^*/*xirp2a*MO mutant/morphant (n = 12/20; 60%)]. These observations did not reveal any indispensable involvement of Xirp1 or Xirp2a in rapid skeletal muscle recovery.

**Figure 10:**
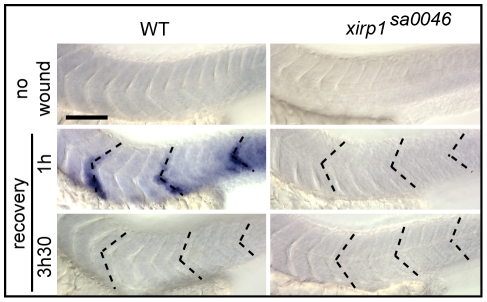
Rapid *xirp1* transcriptional response to myocellular injury. Whole-mount *in situ* hybridizations on 33 hpf embryos which were laser-injured either at 29 hpf (bottom row) or at 32 hpf (middle row) at the level of three different somites (dotted lines). *xirp1* mRNA transcriptional response occurs within one hour after injury and is no longer detectable at 3.5 hours after injury in WT. There is a lack of *xirp1* mRNA expression upon laser-induced myocellular injury in *xirp1^sa0046^* mutants. Scale bar: 100 µm.

To determine the temporal dynamics of *xirp1* transcriptional response to micro-injury, we performed whole-mount *in situ* hybridizations after laser-injury. Whereas in WT *xirp1* expression was detectable already one hour after injury in damaged somitic tissue and persisted for not more than 3.5 hours, expression of the mutant mRNA was completely absent in *xirp1^sa0046^* mutants (Fig. 10). These experiments suggested that either Xirp1 is involved in its own rapid transcriptional regulation upon injury or that nonsense mediated mRNA decay prevents gene expression in *xirp1^sa0046^* mutants. Together, the above experiments showed that Xirp1 expression is strongly responsive to myocellular injury, albeit with unknown function.

### Xirp1 is dispensable for cardiac development and myofibrillogenesis

Functional analysis of chicken cXin had been suggestive of a crucial role in cardiac looping [11]. Because in mouse disruption of either one of two murine Xirp family members, Xirp1 or Xirp2, expressed in the heart did not cause early cardiac defects, it had been suggested that these two Xirps function redundantly [18], [21]. Since Xirp1 is the only Xirp protein containing Xin-repeats expressed in embryonic zebrafish cardiac tissue and since redundancies with other Xirps could therefore be excluded, we assessed its developmental role during cardiac morphogenesis. However, neither early steps of cardiac morphogenesis nor myofibrillogenesis were affected in *xirp1^sa0046^* mutants (Fig. 6B; Fig. 7A). Furthermore, homozygous *xirp1^sa0046^* mutants raised to adulthood were fully viable [survival by 17 months of age: WT, (n = 62/96; 64.6%); *xirp1^sa0046^*, (n = 11/17; 64.7%)] and undistinguishable from WT. Immunohistochemical analyses confirmed that neither Xirp2a nor Xirp2b were upregulated to compensate for lack of Xirp1 within hearts of embryonic or adult *xirp1^sa0046^* mutants (Fig. 6A,C; Fig. 7B). Therefore, unlike reported for chicken, Xirps are not essential for zebrafish cardiac morphogenesis.

**Figure 11:**
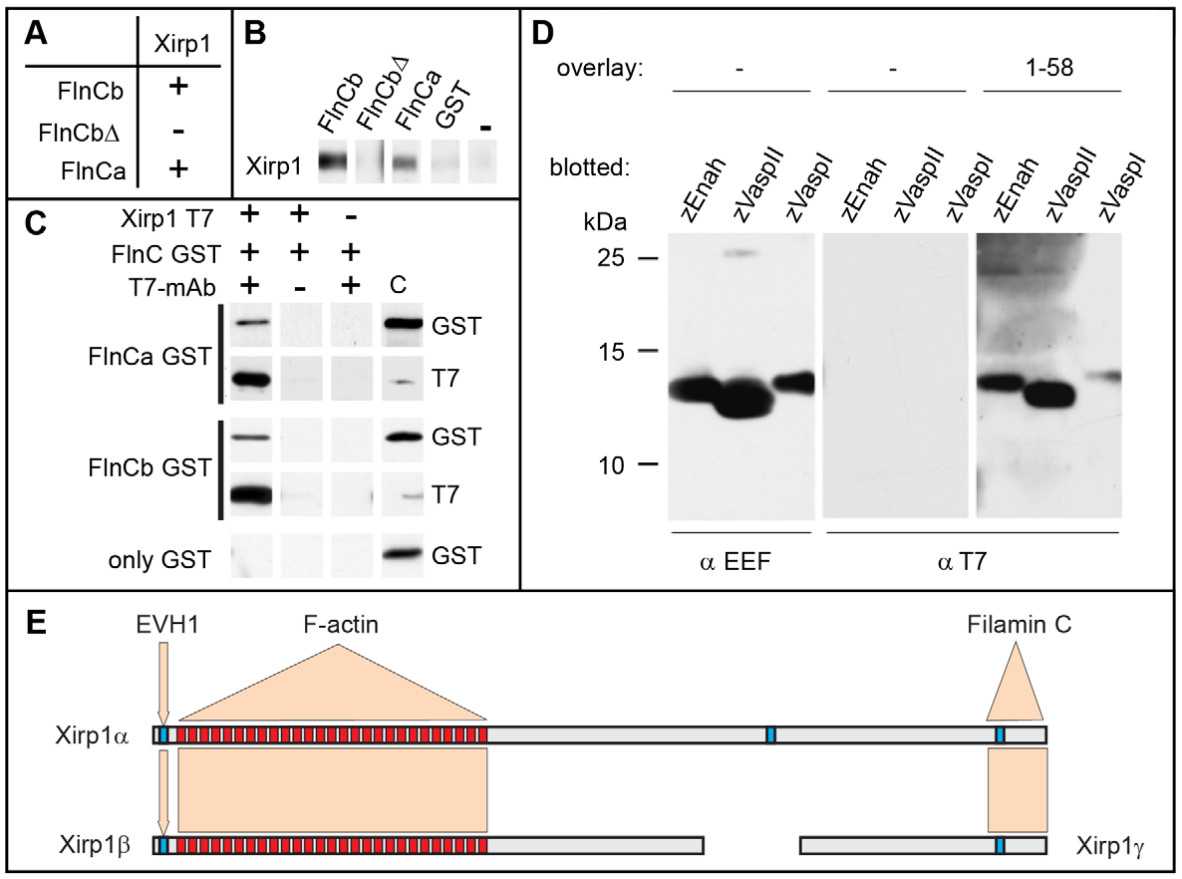
Characterization of interactions between Xirp1 and Filamin C or Enah/Vasp family members. (A) Table summarizing the results of direct yeast two hybrid experiments with the C-terminus of zebrafish Xirp1 (residues 2097–2297) and the GST-tagged domains 19–21 of zebrafish Filamin Ca (FlnCa) and b (FlnCb). The Filamin CbΔ (FlnCbΔ) isoform represents a splice variant lacking the unique insertion within domain 20 that is present in all Filamin C proteins. (B) Western blot overlay experiments show specific binding of the GST-tagged domains 19–21 of FlnCa and FlnCb to the blotted C-terminus of Xirp1. GST and FlnCbΔ do not bind, confirming that the unique insertion within domain 20 of Filamin Cb is necessary for the interaction. (C) Co-immunoprecipitation experiments confirm these interactions: FlnCa and FlnCb are co-precipitated, whereas GST alone is not. (D) Western blot overlay experiments show specific binding of T7-tagged N-terminus of zebrafish Xirp1 (residues 1–58) containing proline-rich repeat1 (PR1) to the blotted EEF-tagged EVH1 domains of zebrafish Enah, VaspI and VaspII. (E) Summary of binding assays shows the isoforms of Xirp1 identified in our study, and the respective binding sites for EVH1 domains of Enah and Vasp (PR1) and Filamin C (carboxy-terminus). Binding to F-actin is presumably mediated by Xin-repeats (red boxes). PRs are depicted by blue boxes.

This unexpected result prompted us to further investigate potential roles of Xirps during the development of other myocellular subtypes. To elucidate potentially redundant roles of all zebrafish Xin-repeats containing proteins in this process, we analyzed skeletal muscle development in *xirp1^sa0046^*/*xirp2a*MO mutant/morphants prior to 24 hpf since Xirp2b is not yet expressed at this stage. Indeed, *xirp1^sa0046^*/*xirp2a*MO mutant/morphant embryos lacked expression of all three Xirps prior to 24 hpf while displaying normal somitic muscle development and correctly striated myofibrils (Fig. 7C,D). Together, these findings suggest that zebrafish Xirps, irrespective of their early developmental expression and their evolutionarily conserved interactions with the sarcomeric and actin-regulatory proteins Filamin C, Enah and Vasp (Fig. 11), are not essential myocellular developmental or repair factors.

## Discussion

In summary, we have outlined evidence for rapid recovery from myocellular injury within the zebrafish embryo which occurs within hours of injury and which does not involve progenitor cell proliferation. Precise laser-induced injuries, as well as pharmacologically induced muscle damage, caused a strong upregulation of Xirp1 expression within injured tissue. We could also show that the ectopic localization of Xirp1 and Xirp2a within damaged cells is a cell-autonomous process that is different from stimulating a progenitor cell proliferation program. However, since many wounds are not entirely closed after 6 hours, additional cell proliferation may also be necessary for complete wound healing. Laser- and pharmacologically-induced injuries can provoke varying degrees of damage, ranging from disrupted myofibrils to complete destruction of the cell. This in turn means that injured cells may be removed, replaced by neighboring cells, or cell-autonomously fully recover from damage. Up to date, the mechanisms underlying the healing of injured muscles are not understood in detail and we provide here first evidence for a rapid repair mechanism which may or may not suffice for complete recovery, depending on the severity of the injury. We propose that Xirp1 is a conserved marker for myocellular injury, not just during embryonic development, since it is upregulated within murine tissue upon excessive exercise or injury as well [12], [14], [15], [16]. However, it is not known whether Xirp1 expression within adult murine myocellular tissues marks a repair process. One difference with our observations is that expression of murine Xirp1 has also been shown in Pax7^+^ muscle progenitor cells [15]. The fact that Xirp1 is strongly upregulated in such various models of muscle damage, hints at a function of Xirp1 during muscle repair. In our assays of injured zebrafish embryos, we found that Xirp1 is dispensable for rapid repair; however, we cannot exclude that Xirp1 has an active function in other contexts of muscle regeneration (e.g. in adult tissues, or when stem cell proliferation is involved). Further functional long-term assessments of the role of Xirp1 in different disease or injury conditions such as muscular dystrophy models are warranted.

Our work in a lower vertebrate has clearly demonstrated that cardiac morphogenesis is normal in embryos that completely lack all Xirps within cardiac tissue. Notably, our findings are unexpected given that functional studies in chicken had implicated Xirps in cardiac morphogenesis and in the evolution of the vertebrate multi-chambered heart [11], [23]. In contrast, our findings and studies on the role of murine Xirps [18], [21] now raise serious doubts about the relevance of Xirps for cardiac morphogenesis in any vertebrate species. Murine Xirp knock-out models lack obvious developmental defects and develop mild cardiac phenotypes as adults [18], [19], [20], [21] which is consistent with our finding that zebrafish Xirp proteins are not required for early cardiac morphogenesis. It has been suggested that mice lacking all Xirp proteins in the heart would have more severe cardiac defects than single Xirp knock-outs. However, we could not detect any cardiac defects in zebrafish adult *xirp1^sa0046^* mutants that entirely lack cardiac Xirps.

Under normal conditions, Xirp proteins localize to the MTJ, a site of myofibrillar growth which involves actin remodeling. Since Xirp1 and Xirp2a are also ectopically present at sites of muscle injury, Xirp proteins may have in common a high affinity for non-striated actin-rich myofibrils or their binding proteins. This subcellular distribution and the direct association of Xirps with other actin-regulatory proteins suggest that Xirp proteins may contribute to the organization of actin filaments, together with other unidentified proteins. We could verify the evolutionary conservation of interactions with several conserved sarcomeric or actin-regulatory binding partners (Fig. 11). These analyses suggest that zebrafish Xirp1 may be involved in conserved protein-protein interactions at the ICD or the MTJ. Even though a functional involvement of Xirp1 in cardiac and skeletal muscle development or in myocellular repair is speculative, we could show that Xirp1 is rapidly responsive to myocellular injury. What determines this transcriptional response also awaits further clarification. Further characterization of the molecular signatures during normal myocellular repair will be informative for human cardiac or skeletal muscle repair.

## Materials and Methods

### Fish stocks and maintenance

General zebrafish maintenance and embryo collection was carried out according to standard conditions. The characterization of adult *xirp1* mutant fish was performed with permission by the local authorities (Senatsverwaltung für Gesundheit, Umwelt und Verbraucherschutz, Berlin [Lageso Berlin]) under animal experimentation project G0069/10. Embryos were staged at 28.5°C. The following fish strains were used: AB/WIK, TüLF/WIK, Tg[*myl7:gfp*] [28]. The *xirp1^sa0046^* allele was obtained from the Wellcome Trust Sanger Center (Cambridge, UK). Mutant fish were genotyped and sequenced using the following primer pair:

sa0046-fwd: 5′-TTTAGGTCGCTGCTGCTCTGTCGAA-3′

sa0046-rev: 5′-ACACCTTCGTGAATTGCATCAAGCG-3′

### Pharmacological treatment of zebrafish embryos

Cell proliferation was determined by BrdU labeling according to established conditions using 5 mg/ml BrdU in E3 medium (B5002, Sigma) [29]. We used 150 µM Aphidicolin in E3 medium (Sigma, A0781) to block cell proliferation [26]. Both treatments started at 24 hpf followed by wounding of embryos at 31 hpf and recovery in BrdU or BrdU+Aphidicolin solutions for 2 hours at 28.5°C.

For scoring wound healing depending on cell proliferation, wild-type embryos were incubated with or without 150 µM Aphidicolin from 24 hpf onwards, laser-injured at 28 hpf and left to recover in with or without Aphidicolin until 34 hpf (6 h recovery), or wounded at 33.5 hpf and fixed immediately (no recovery) for further analysis. Each embryo was tracked individually: after wounding and imaging of the wounds, each embryo was kept separately to be imaged again at a later timepoint. Actin staining of the embryos with rhodamine phalloidine was used to visualize myofibrils and detect open wounds. Large laser injuries were inflicted in two distinct somite blocks in each fish and scored separately. Numbers of open, visible wounds/number of injuries after 6 hours recovery were counted and an unpaired t-test was performed to analyze the statistical significance of Aphidicolin treatment on wound closure. There is no significant difference whether with or without recovery: p-value 0.2149 (with 6 hours recovery), p-value 0.6985 (without recovery period).

To induce myocellular injury, zebrafish embryos were incubated in 1 mM Gal (Enzo life sciences, ALX-550-336-M050) in E3 medium between 80% epiboly and 2 or 3 dpf [22].

### Microarray analysis

Microarray analysis, data preprocessing, quality control, transformation, and normalization were performed as previously described [30]. All data is MIAME compliant; the raw data with the accession number GSE30729 has been deposited in the public repository GEO at NCBI.

### Laser-induced myocellular injuries

Tricaine anesthetized embryos were wounded and then left to recover in E3 medium at 28.5°C for different times and finally fixed and immunostained for analysis. A micropoint laser connected to a Zeiss Axioplan was used for injuring according to manufacturers' instructions using a 20× objective and a laser pulse at a wavelength of 435 nm for cell ablation (Photonics, Inc.). Timelapse recordings were generated using a camera mounted on a Zeiss Axioplan; wounding and recording was performed using a 20×/dry and a 40×/oil objective, respectively.

### Zebrafish Morpholino antisense-oligonucleotide microinjection

The *xirp2a* splice-blocking MO (Gene-Tools) was injected at a concentration of 200 µmol/L:

MO*^xirp2a^*: 5′-CTTTATTTTTACCTTGATATGTTTG-3′

### Generation of antibodies and immunohistochemistry

For the immunization of rabbits, parts of the large exon of all zebrafish *xirp* genes encoding different fragments [Xirp1: (P43; Xirp1α/β) aa1117–1422, (P47; Xirp1α/γ) aa2097–2297; Xirp2a: aa2974–3276; Xirp2b: aa1697–2049] were amplified from adult zebrafish cDNA using *Pfu* polymerase [31]. Proteins were expressed and purified as described [32]. Rabbits were immunized four times with the purified recombinant proteins according to a standard protocol (Biogenes, Berlin, Germany). Antibodies were purified as described [32]. Immunohistochemistry was performed as described, with an Aceton permeabilization step of 5–10 minutes at −20°C prior to the blocking step [29]. For tissue sectioning of Xirp immunostainings, transverse sectioning was performed according to [29]. Unless stated otherwise, Xirp1 immunostainings were performed with the P43 antibody which detects Xirp1α/β, as they are the dominantly expressed isoforms. To detect Xirp1α/γ, P47 staining was performed upon very mild fixation (4% PFA for 30 min). The following antibodies were used: rabbit anti-Xirp1α/β (1∶500; P43), rabbit anti-Xirp1α/γ (1∶500; P47), rabbit anti-Xirp2a (1∶500), rabbit anti-Xirp2b (1∶500), mouse anti-β-catenin (1∶500, Sigma), mouse anti-Alcam (1∶500, zn-8, DSHB), mouse anti-Titin A/I (1∶10; D.O. Fürst), mouse anti-α-Actinin (1∶500; Sigma), mouse anti-Myosin (1∶10; S46 and F59, both DSHB), mouse anti-Tropomyosin (1∶10; DSHB), mouse anti-Pax7 (1∶10; DSHB), mouse anti-BrdU (1∶100; Roche Diagnostics), goat anti-mouse FITC (1∶200; Jackson), goat anti-mouse Cy5 (1∶200; Jackson), goat anti-rabbit FITC (1∶200; Jackson), goat anti-rabbit Cy5 (1∶200; Jackson), rhodamine phalloidin (1∶100; Invitrogen). Recordings of immunostained tissues were performed at the LSM Meta510 FSC or LSM Meta510 NLO (Zeiss) with 40×/oil or 100×/oil objectives. Images were analyzed with the LSM image browser (Zeiss) and processed using Image J (Rasband, W.S., ImageJ, U.S. National Institutes of Health, Bethesda, Maryland, USA, http://rsb.info.nih.gov/ij/, 1997–2008) or Adobe Photoshop (Adobe).

### Analysis of the gene structure of the *xirp1*, *xirp2a* and *xirp2b* genes

Databases (http://genome.ucsc.edu; www.ensembl.org, http://zfin.org) were searched for sequences homologous to the consensus sequence encoding Xin-repeats as they are found in mammalian and chicken Xirps. To identify splice variants of *xirp1*, EST databases were examined for the presence of sequences corresponding to the genes. EST sequences were aligned with the genome using BioEdit software in order to identify intron and exon sequences.

### RT-PCR and 5′ RACE

Gene predictions were verified by RT-PCR and 5′RACE experiments. For both types of experiments, total RNA was purified from 24 hpf zebrafish embryos using the RNeasy mini kit (Qiagen, Hilden, Germany). The Superscript One-Step RT-PCR Kit with Platinum Taq was used according to the manufacturers' suggestions (Invitrogen) for products of approximately 1000 bp or less. For the analysis of longer fragments, cDNA was synthesized using either a gene-specific primer or random nonamers, and M-MLV Reverse Transcriptase RNase H Minus, according to the instructions of the manufacturer (Promega, Mannheim, Germany).

5′ RACE was performed using the 5′ RACE System (Invitrogen). cDNA was synthesized using primers corresponding to a site close to the 5′ end of the large exon of the *xirp* genes. A further primer corresponding to a site more downstream within this exon was used to perform nested PCR. All amplicons were cloned into the pGEM-T vector (Promega, Mannheim, Germany) and sequenced (LGC, Berlin, Germany).

### Whole-mount *in situ* hybridization

Probes for whole mount *in situ* hybridizations were amplified from genomic zebrafish DNA using the following primers:

xirp1-fwd 5′-CTTGAAGAAGCTCAGAGAGGCG-3′

xirp1-rev 5′-TTGGCCAGAATCGGCTTGATCTG-3′

xirp2a-fwd: 5′-CTCAGCAGAGCACGGTGGAAAAC-3′

xirp2a-rev: 5′-GATGGGGCGGGTTTCAAACATCC-3′

xirp2b-fwd: 5′-CTGGAGCAGATCAGAGAGGAGAG-3′

xirp2b-rev: 5′-GCTCACCACGTCAGAAGACTGGG-3′

All amplicons were cloned in pGEMT and sequenced. Whole mount *in situ* hybridizations were performed as previously described [33]. For documentation, stained embryos were cleared in benzyl∶benzoate (2∶1) and embedded in Permount. Images were recorded on a Zeiss Axioplan microscope with 10× objective by using a SPOT digital camera (Diagnostic Instruments) and Meta Morph software (Visitron). Images were processed with Photoshop software (Adobe).

### Cloning of Expression Constructs

To generate the plasmids *βactin:α-actinin-gfp*, *βactin:xirp1γ-gfp* and *βactin:xirp1γ-ires-gfp*, human *ACTININ2* and zebrafish *xirp1γ* were subcloned into the Gateway pDONOR 221 vector (Invitrogen,USA) using the following primers containing flanking attB1 and attB2 sites:

actinin2-fwd: 5′-GGGGACAAGTTTGTACAAAAAAGCAGGCTATGAACCAGATAGAGCCCGGCGTG-3′

actinin2-rev: 5′-GGGGACCACTTTGTACAAGAAAGCTGGGTCCAGATCGCTCTCCCCGTAGAG-3′

xirp1γ-fwd: 5′-GGGGACAAGTTTGTACAAAAAAGCAGGCTCCAGAAGTGAGCATCCAACCAGC-3′

xirp1γ-rev: 5′-GGGGACCACTTTGTACAAGAAAGCTGGGTGATGCTTTTCAGTGTTGGGATGACCAA-3′

xirp1γstop-rev: 5′-GGGGACCACTTTGTACAAGAAAGCTGGGTTTAATGCTTTTCAGTGTTGGGATGACCAA-3′

For generation of the *βactin:xirp1γ^ΔFilCBD^-gfp* construct, the attB1-xirp1γ-attB2 intermediate was PCR amplified using the following primer pair, digested with AvrII and religated upon itself, thereby creating a deletion within Xirp1γ:

xirp1γ^ΔFilCBD^-fwd: 5′-CGTCCTAGGAGGTCACTAATACCATCTAAGTAGTTG-3′

xirp1γ^ΔFilCBD^-rev: 5′-GCACCTAGGAGCAGGCTTTGTTTTGCTGGATAC-3′

In subsequent steps, these plasmids were recombined with destination vector pDEST Tol2pA (gift of N. Lawson), vector p5E-β-actin2 (gift of C.-B. Chien) containing the *β-actin* promoter, vector p3E-EGFPpA (gift of C.-B. Chien) encoding a C-terminal EGFP tag, or vector p3E-IRES-EGFPpA encoding an IRES GFP cassette (gift of C.-B. Chien).

### Expression Constructs and Purification of Recombinant Proteins

cDNA fragments covering domains 19-21 of Filamin Ca and Filamin Cb, were cloned into pGEX6P3 (Amersham Biosciences), and the carboxy-terminus of Xirp1 into pET23-T7. Constructs were transformed to the *E. coli* strain BL21-CodonPlus(DE3)-RP (Stratagene). Expression and purification were carried out essentially as described [34]. Protein concentrations were determined as described [34].

### Western Blot Overlay and Co-Immunoprecipitation Assays

Extracts of bacterial cells expressing the recombinant polypeptide Xirp1 aa2097–2297 were used for SDS-PAGE using 12% polyacrylamide gels, and separated proteins were transferred to a nitrocellulose membrane. Nitrocellulose strips were either incubated with a tag-specific antibody and a HRP-conjugated secondary antibody to detect the expressed polypeptide, or overlaid with recombinant polypeptide (GST-tagged Filamin C fragments). Bound protein was immunodetected using an anti-GST antibody (Santa Cruz), HRP-conjugated secondary antibodies (Jackson Immuno Research Laboratories, Soham, UK), and ECL using “SuperSignal West Pico Chemiluminescent Substrate” (Pierce, Rockford, IL, USA) and Kodak XAR-351 film. For co-immunoprecipitation experiments the purified bacterially expressed T7 and His6-tagged carboxy-terminus of Xirp1 was mixed with purified recombinant GST-tagged Filamin C fragments. T7 antibody was added to the mixture and complexes were immunoprecipitated using ProteinG-coupled Dynabeads. Immunoprecipitated proteins were separated by PAA gel ectrophoresis and blotted to nitrocellulose. The presence of the recombinant proteins on the blots was analyzed using anti T7-tag or anti-GST antibodies using standard procedures.

### Yeast two hybrid assays

To investigate whether the carboxy-terminus of Xirp1 interacts with Filamin C, the cDNA fragment of Xirp1 encoding residues aa2097–2297 was cloned into the pLexA vector. Fragments homologous to human Filamin C domains 19–21 (the Filamin C fragment that interacts with human Xirp) from both zebrafish Filamin Ca/b cDNAs, were amplified by RT-PCR using total RNA isolated from 24 hpf zebrafish embryos as a template. All cDNA fragments were cloned into the pAct2 vector, sequenced and cotransformed with the pLexA vector with the cDNA fragment derived from Xirp1. Growth on SD-LWH agar plates and activity of ß-galactosidase were assayed as described [35].

## Supporting Information

Movie S1 **Live laser wounding assay.** Live stream acquisition of the laser wounding assay within 28 hpf zebrafish skeletal muscle. The two laser pulses are marked (yellow circle) and the retraction of myofibrils at both ends is indicated by red arrows. Changes also occur within neighboring cells.
(MP4)
